# Putting the Barker Theory into the Future: Time to Act on Preventing Pediatric Obesity

**DOI:** 10.3390/ijerph13111151

**Published:** 2016-11-17

**Authors:** Angelo Pietrobelli, Massimo Agosti, Gianvincenzo Zuccotti, the MeNu Group

**Affiliations:** 1 Pediatric Unit, Università degli Studi di Verona, Piazzale L.A. Scuro 10, 37134 Verona, Italy; 2 Pennington Biomedical Research Center, Baton Rouge, LA 70808, USA; 3 Neonatology and Neonatal Intensive Care Unit, Maternal & Child Department del Ponte Hospital, Azienda Ospedaliera di Circolo Fondazione Macchi, 21100 Varese, Italy; massimo.agosti@ospedale.varese.it; 4 Department of Pediatrics, Ospedale dei Bambini “V. Buzzi”, Director of the Center for Research on Nutrition (CURN), Biomedical and Clinical Science Department, Università degli Studi di Milano, 20157 Milan, Italy; gianvincezo.zuccotti@unimi.it

**Keywords:** nutrition, prevention, pediatric obesity, growth, first 1000 days

## Abstract

Growth and development are key characteristics of childhood and sensitive markers of health and adequate nutrition. The first 1000 days of life—conception through 24 months of age—represent a fundamental period for development and thus the prevention of childhood obesity and its adverse consequences is mandatory. There are many growth drivers during this complex phase of life, such as nutrition, genetic and epigenetic factors, and hormonal regulation. The challenge thus involves maximizing the potential for normal growth without increasing the risk of associated disorders. The Mediterranean Nutrition Group (MeNu Group), a group of researchers of the Mediterranean Region, in this Special Issue titled “Prevent Obesity in the First 1000 Days”, presented results that advanced the science of obesity risk factors in early life, coming both from animal model studies and studies in humans. In the future, early-life intervention designs for the prevention of pediatric obesity will need to look at different strategies, and the MeNu Group is available for guidance regarding an appropriate conceptual framework to accomplish either prevention or treatment strategies to tackle pediatric obesity.

Growth and development are key characteristics of childhood and sensitive markers of health status and adequate nutrition. Infants with restricted intrauterine growth are more likely to have poor cognitive development during childhood, and they are also at increased risk of cardiovascular, pulmonary, and kidney disease in later life [1]. The first 1000 days of life—conception through age 24 months—represent a fundamental period for development; thus, the prevention of childhood obesity is quite important [2]. In recent years, the World Health Organization’s Report by the Commission on Ending Childhood Obesity emphasized the important role of the preconception, antenatal, and early childhood periods in the prevention of childhood obesity [3]. In light of this fact, the Mediterranean Nutrition Group (MeNu), a group of researchers from the Mediterranean region, contribute to the prevention of pediatric obesity through their research and medical activities. Researchers of the MeNu Group, have published articles that advance the science of obesity risk factors in early life and its prevention, from animal model studies to studies on humans. Taken together, the articles in this Supplement entitled “Prevent Obesity in the First 1000 Days,” which investigates modifiable risk factors for childhood obesity that occur in the first 1000 days, indicate strong evidence for risk factors in pregnancy, having observed animal models and infants. Additionally, we examined feeding and lifestyle practices that impacted a child’s growth. We discuss the diet in European Mediterranean countries, keeping in mind the unique role of the Mediterranean diet per se and the importance of human milk as a significant food for growth. We present feeding strategies to facilitate a child’s acceptance of fruits and vegetables, looking at the quality of early nutrition in relation to risks of obesity later in life. We also present ten good practices that have been shown to have a beneficial effect on children’s growth, suggesting specialized counseling focused on changing the behavior of an individual or of a whole family.

Early-life intervention designs in the future for the prevention of pediatric obesity will need to look at all of the above-mentioned strategies, and the MeNu Group is available for guidance regarding an appropriate conceptual framework to accomplish these complex aims of prevention.
